# Barriers and facilitators to the delivery of rehabilitation in health systems by chiropractors: a SWOT analysis of perspectives from a world federation of chiropractic congress workshop

**DOI:** 10.1186/s12998-026-00660-0

**Published:** 2026-07-01

**Authors:** Jordan A. Gliedt, Afua Adjei-Kwayisi, Nora Bakaa, Jean-Luc Gauthier, Brett Guist, Katie de Luca

**Affiliations:** 1 Department of Neurosurgery, Medical College of Wisconsin, Milwaukee, WI USA; 2 Physical Therapy and Rehabilitation Department, Greater Accra Regional Hospital, Accra, Ghana; 3 School of Physical Therapy, Western University, London, ON Canada; 4 Département de Chiropratique, Université du Québec À Trois-Rivières, Trois-Rivieres, Canada; 5 Canadian Memorial Chiropractic College, Toronto, ON Canada; 6 Discipline of Chiropractic, School of Health, Medical and Applied Sciences, CQUniversity, Brisbane, Australia

**Keywords:** Rehabilitation, Disability, Chiropractic, Global health, SWOT analysis

## Abstract

**Background:**

Global unmet need for rehabilitation remains substantial. Chiropractors commonly manage musculoskeletal conditions that may benefit from rehabilitation. However, barriers and facilitators to chiropractors’ delivery of rehabilitation in health systems have yet to be well described. Therefore, this study aimed to identify local, regional, and global barriers and facilitators to the delivery of rehabilitation within health systems.

**Methods:**

Data were collected from participants attending the 18th World Federation of Chiropractic (WFC) Biennial Congress, held in Copenhagen, Denmark, on May 10, 2025. A deductive, manifest-directed content analysis, framed by the Strengths, Weaknesses, Opportunities, and Threats (SWOT) framework, was conducted on responses collected during a rehabilitation workshop. Participants completed free-text responses to four SWOT-based prompts on the delivery of rehabilitation by chiropractors within health systems. Responses were categorized into SWOT domains and coded according to an adaptation of predefined barriers and facilitators to rehabilitation delivery.

**Results:**

A total of 369 responses were recorded and catalogued into strengths (n = 89), weaknesses (n = 103), opportunities (n = 84), and threats (n = 93). Commonly reported strengths included education, clinical, and leadership and policy categories. Weaknesses were primarily related to categories of facilities/logistics, finances, education, and leadership and policy. Opportunities primarily included facilities/logistics, leadership and policy, and education categories. Threats were primarily related to leadership and policy, cultural, and political categories.

**Conclusion:**

This study highlights local, regional, and global factors influencing the delivery of rehabilitation in health systems by chiropractors. Findings underscore the need for targeted implementation strategies, policy advocacy, and expanded professional training to optimize chiropractors' contributions to the global rehabilitation workforce.

**Supplementary Information:**

The online version contains supplementary material available at https://doi.org/10.1186/s12998-026-00660-0.

## Introduction

According to the World Health Organization (WHO), 2.4 billion individuals worldwide live with a health condition that could benefit from rehabilitation [1]. Though unmet rehabilitation needs are widespread throughout the world, disparities in access to rehabilitation in low- and middle-income countries (LMICs) are startling. Up to 50% of individuals living in LMICs do not have access to rehabilitation services [1]. Unmet rehabilitation needs are expected to continue to increase worldwide due to factors such as limited access to rehabilitative services, insufficient funding and/or policy prioritization, ineffective or underutilized referral pathways to rehabilitation, and/or a shortage of a sufficiently trained rehabilitation workforce [1].

Chiropractors are trained in assessment, diagnosis, treatment, and prevention of musculoskeletal disorders, [2] conditions that are the leading cause of years lived with disability worldwide [3–5] and commonly benefit from rehabilitation [6]. Upon consideration of WHO’s definition of rehabilitation as “a set of interventions designed to optimize functioning and reduce disability in individuals with health conditions in interaction with their environment”, chiropractors use a variety of modalities during treatment and for the prevention of musculoskeletal disorders, which are encompassed under this umbrella [2, 7].

Globally, many people seek healthcare from chiropractors, with the median 12-month utilization of chiropractic services measured at 9.1% [8]. There are higher estimates of utilization among people living with chronic low back pain (14.5%), [9] which is a leading cause of disability globally [3]. With the world facing a chronic shortage of health care workers for rehabilitation, the chiropractic profession must make concerted efforts to identify the barriers and facilitators to the delivery of rehabilitation by chiropractors within health systems.

As part of WHO’s effort to construct a global rehabilitation competency framework to support greater access to rehabilitation services, the World Federation of Chiropractic (WFC) has begun efforts to develop and guide chiropractic education and direct rehabilitation policies across local and regional jurisdictions globally [10]. This framework includes several competencies across three domains of rehabilitation: (1) basic concepts of rehabilitation and disability, (2) legal, regulatory and ethical components, and (3) rehabilitation management of disability and other health conditions.

Despite the chiropractic profession’s efforts to advance rehabilitation delivery, limitations due to political influences, regulatory barriers and socioeconomic and sociodemographic inequities still exist [11, 12]. We are unaware of any prior studies that have explored potential barriers and facilitators to chiropractors delivering rehabilitation in local, regional and global health systems. Therefore, the purpose of this study was to explore the barriers and facilitators to the delivery of rehabilitation by chiropractors in health systems worldwide.

## Methods

### Study overview and setting

**Fig. 1:**
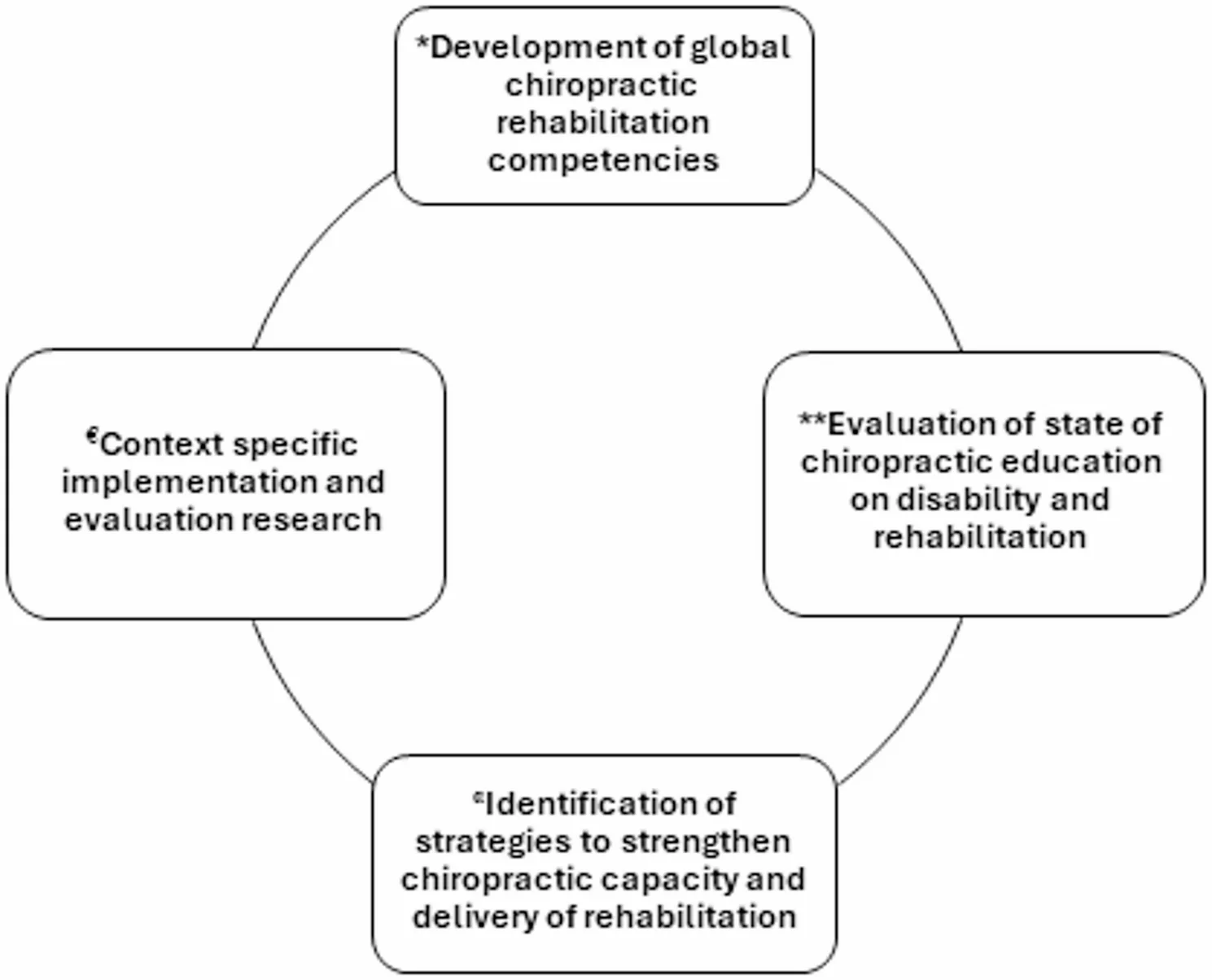
Phases of research agenda for the evaluation and advancement of chiropractic education and delivery of rehabilitation. *World Federation of Chiropractic (WFC) rehabilitation competency framework completed^9^; **Evaluation currently being planned as part of a separate study by research team; ^**α**^Current study described in this document; ^**€**^Future studies necessary

This study was approved by the Medical College of Wisconsin, Institutional Review Board (PRO00055146). This study is reported in accordance with the Standards for Reporting Qualitative Research (SRQR) [13]. This study focuses on strategies to strengthen the capacity of chiropractors as part of the global rehabilitation workforce, which is one aspect of the WFC’s larger research agenda that evaluates chiropractic education and the delivery of rehabilitation (Fig. 1).

The setting for this study was a rehabilitation workshop for chiropractors, academic chiropractic professionals, and chiropractic stakeholders in attendance at the 18th World Federation of Chiropractic (WFC) Biennial Congress in Copenhagen, Denmark, on May 10, 2025. The WFC Biennial Congress is the “only international forum for chiropractic national association leaders and executives worldwide to share their practice experience, develop networks and learn about new areas of innovation from other national associations” [14]. The WFC has national association members from more than 90 countries across each of the major world regions. Therefore, the WFC Biennial Congress was an ideal setting for data collection, as it attracts a global audience of chiropractors, academics, educators, students, policymakers, and community members.

A 90 min workshop titled “Chiropractors as part of the rehabilitation revolution: Identifying strategies to strengthen rehabilitation in local, regional and global health systems” was led by members of the study team (JAG, AAK, KD). Attendance at the workshop was voluntary and not required for attendance at the WFC Congress. The workshop program consisted of two components: (1) a 30 min education session on the WFC global chiropractic rehabilitation competency framework, [10] and (2) a 45 min session to identify barriers and facilitators to the local, regional, and global delivery of rehabilitation in health systems.

### Target population

The target population included chiropractors, academics, chiropractic students, chiropractic educators, policymakers, and other chiropractic community members who were currently or prospectively interested in the delivery of rehabilitation in health systems by chiropractors.

### Eligibility criteria

Inclusion criteria included: (1) in-person, live attendance at the aforementioned workshop; (2) self-reported ability to understand and communicate in English-language in verbal and written form; and (3) self-report of any of the following: an individual who has completed a chiropractic degree program at an institution accredited by a governmentally recognized accreditation agency, an individual who is currently an enrolled student at a chiropractic degree program that is accredited by a governmentally recognized accreditation agency, a policymaker, or other individual who self identifies as an active member of the chiropractic community who is currently or prospectively interested in chiropractic delivery of rehabilitation services.

### Recruitment and data collection

Attendees of the workshop were verbally invited to participate in this study. An electronic participant information letter was presented visually on a projection screen for workshop attendees. The participant information letter was also read aloud to attendees during the workshop, prior to data collection. Participation was voluntary and completely anonymous. Respondents had the option to participate or opt out of any part of this study. No incentives were provided to respondents. Demographic characteristics of respondents were not collected.

Based on the Strengths, Weaknesses, Opportunities, and Threats (SWOT) framework, 4-question prompts were developed for respondents to provide free-text responses (Table 1). We used the SWOT framework to understand barriers and facilitators to the local, regional, and global delivery of rehabilitation by chiropractors within health systems. The SWOT framework is widely used in human resource development and can aid policy and decision-making across sectors [15, 16]. Further, the SWOT framework is advantageous for identifying internal (e.g., strengths and weaknesses) and external factors (e.g., opportunities and threats) that impact chiropractic rehabilitation delivery in health systems, as well as future avenues for implementation [17–19].

**Table 1:** 4-question prompts which respondents provided free text responses

Question prompts
What are core strengths (e.g., unique knowledge, talent, assets, resources, capabilities, etc.) of chiropractors/chiropractic profession in delivering rehabilitation services in health systems? *Consider Local, Regional/National, and Global perspectives
What are core weaknesses (e.g., disadvantages, knowledge or skill deficits, etc.) of chiropractors/chiropractic profession in delivering rehabilitation services in health systems? *Consider Local, Regional/National, and Global perspectives
What are core opportunities (e.g., unmet societal or health system needs, strengths leveraged to opportunities, etc.) of chiropractors/chiropractic profession in delivering rehabilitation services in health systems? *Consider Local, Regional/National, and Global perspectives
What are core threats (e.g., obstacles, weaknesses that are preventing service delivery, policy/legislation challenges, etc.) of chiropractors/chiropractic profession in delivering rehabilitation services in health systems? *Consider Local, Regional/National, and Global perspectives

During data collection, for each question prompt, attendees were provided with approximately 10 min to record free-text responses. Respondents were not limited in their free-text responses and were encouraged to provide any number of responses to each prompt at the detail and length of their choosing. Responses were anonymously recorded on paper and collected by the study team (JAG, AAK, KD).

Responses were transcribed verbatim into a single Microsoft Word document (JAG, KD), uploaded to MAXQDA, and stored on a password-protected server at the Medical College of Wisconsin.

### Data analysis

Because prior literature had already identified barriers and facilitators relevant to rehabilitation service delivery, [20] we used a deductive content analysis at the manifest level [21]. Consistent with this methodology, overarching SWOT domains (strengths, weaknesses, opportunities, threats) were identified a priori. The data analysis was predominantly performed by the primary investigator (JAG).

Participant responses were first sorted into the a priori SWOT domains, consistent with the structure of the four workshop prompts. Thereafter, a deductive approach to coding was applied to all sorted data, using a chiropractic-context adaptation of predefined barriers and facilitators to the delivery of rehabilitation, as described by Htwe et al. [20] Table 2 summarizes the coding matrix for this analysis. The analysis focused on the explicit (manifest) content of responses [22]. Results of the data analysis were discussed with the study team, and any disagreements were resolved through discussion and consensus. A separate inductive coding phase to generate new categories or subthemes from the data was not completed.

**Table 2:** Predefined categories, subcategories, and barriers and facilitators to rehabilitation coding matrix

SWOT category	Barriers and facilitators to chiropractic delivery of rehabilitation in health systems
Strengths	Finances
	Facilities/logistics
	Education
	Resources
	Leadership and policy
	Technology/advanced treatment
	Community-based rehabilitation
	Social support
	Cultural
	Political
	Clinical
	Registries and standards
Weaknesses	Finances
	Facilities/logistics
	Education
	Resources
	Leadership and policy
	Technology/advanced treatment
	Community-based rehabilitation
	Social support
	Cultural
	Political
	Clinical
	Registries and standards
Opportunities	Finances
	Facilities/logistics
	Education
	Resources
	Leadership and policy
	Technology/advanced treatment
	Community-based rehabilitation
	Social support
	Cultural
	Political
	Clinical
	Registries and standards
Threats	Finances
	Facilities/logistics
	Education
	Resources
	Leadership and policy
	Technology/advanced treatment
	Community-based rehabilitation
	Social support
	Cultural
	Political
	Clinical
	Registries and standards

*Finances* costs of services/assistive devices/medications/home visits/telehealth, patient socioeconomic status, insurance, payment/reimbursement for delivery of rehabilitation services, source of rehabilitation service delivery

*Facilities/logistics* infrastructure and accessibility of rehabilitation for patients (e.g., transportation, distance, etc.), availability of chiropractic rehabilitation services and devices, fragmented rehabilitation services, employment opportunities for chiropractors, electronic health records integration, integration of chiropractic services in health systems, chiropractic workforce capacity

*Education* professional development/continuing education for chiropractors on rehabilitation, research and information capacity, disability knowledge, rehabilitation and disability literacy among chiropractors and patients, education in accessible languages, research skills of chiropractors, career progression opportunities for chiropractors, chiropractic telehealth acceptance/support, use of evidence-based practices, rehabilitation knowledge and skills of chiropractic workforce, professional organization support for chiropractors

*Resources* patient access to rehabilitation services and aids, clinical staff support, suitable healthcare facilities, health system support, clinician time, clinician access to research and information outside of academia

*Leadership and policy* political will, government support for rehabilitation delivery and research, governmental leadership, organizational mandates, legislation, strategies to address affordability, unavailability of regulatory bodies, coordination between organizations and government, health system capacity, political equality for disabled

*Technology/advanced treatment* assistive technology, communication and information in establishing service organizations

*Community-based rehabilitation* availability of programs, research for implementing telehealth

*Social support* healthcare providers and public perceptions of chiropractors, patient and caregiver support, patient telehealth utilization, caregiver pay requests, patient awareness of chiropractic rehabilitation service availability

*Cultural* disability beliefs, religious beliefs and practices, health beliefs, racial prejudice, rehabilitation knowledge and culture, language, personal behavior

*Political* political stability, interprofessional relations

*Clinical* interdisciplinary collaboration, clinical team collaboration, clinical skills and outcomes, protocols, coordination between healthcare sectors

*Registries and standards* disability data, guidelines and accreditation standards, chiropractic discipline definition, definitions and measurement methods, disaster-related competencies, documentation of outcomes, compliance with rehabilitation standards, recognition of chiropractors as rehabilitation professionals

Investigator reflexivity and positionality were considered and disclosed to enhance the trustworthiness of findings [23]. The study team comprises clinicians and researchers, particularly chiropractors, with extensive clinical practice experience in rehabilitation services across LMICs and high-income countries. In addition, multiple investigators hold PhD-level training, including expertise in qualitative methods. The primary investigator and data analyst (JAG) is an experienced clinician with more than 13 years of clinical practice, international service experience in the chiropractic profession, and experience in qualitative data analysis, as well as a doctorate-level training in qualitative methods.

We present our findings in a narrative format. Given the nature of the collected data, which included brief responses, and the ability of a deductive directed content analysis at the manifest level approach to evaluate explicit manifest (surface level) data, we highlight and contextualize our findings with a description of the most frequent predefined categories of barriers and facilitators to rehabilitation delivery that emerged from each of the individual SWOT framework domains. We provide support for our findings with tables and quotations from the data.

## Results

From the workshop, 45 individuals provided 369 written responses to the 4-question prompts. These included 89 responses related to strengths, 103 responses related to weaknesses, 84 responses related to opportunities, and 93 responses related to threats. Table 3 summarizes the frequency of each predefined category of barriers and facilitators to the local, regional, and global delivery of rehabilitation by chiropractors in health systems described by participants.

**Table 3:** Summary of frequency of responses on barriers and facilitators to chiropractic delivery of rehabilitation in health systems, according to the SWOT framework

Strengths	Weaknesses
Education (n = 22)	Facilities/logistics (n = 20)
Clinical (n = 20)	Finances (n = 15)
Leadership and policy (n = 13)	Education (n = 13)
Facilities/logistics (n = 10)	Leadership and policy (n = 13)
Registries and standards (n = 8)	Political (n = 12)
Resources (n = 7)	Social support (n = 9)
Social support (n = 4)	Cultural (n = 8)
Finances (n = 2)	Clinical (n = 6)
Political (n = 2)	Resources (n = 4)
Cultural (n = 1)	Registries and standards (n = 3)
Technology/advanced treatment (n = 0)	Technology/advanced treatment (n = 0)
Community-based rehabilitation (n = 0)	Community-based rehabilitation (n = 0)

Opportunities	Threats
Facilities/logistics (n = 17)	Leadership and policy (n = 18)
Leadership and policy (n = 13)	Cultural (n = 15)
Education (n = 10)	Political (n = 13)
Social support (n = 9)	Facilities/logistics (n = 12)
Political (n = 9)	Finances (n = 12)
Cultural (n = 8)	Social support (n = 10)
Clinical (n = 8)	Education (n = 8)
Registries and standards (n = 4)	Resources (n = 2)
Finances (n = 3)	Clinical (n = 2)
Resources (n = 3)	Registries and standards (n = 1)
Technology/advanced treatment (n = 0)	Technology/advanced treatment (n = 0)
Community-based rehabilitation (n = 0)	Community-based rehabilitation (n = 0)

### Strengths

Education (n = 22), clinical (n = 20), and leadership and policy (n = 13) factors were the most frequently described strengths. Within the education category, multiple responses described strong professional development and education opportunities for chiropractors, including in rehabilitation.

*“Quality [doctor of chiropractic] education and continuing professional development with a focus on person-centred, integrated evidence-based care”*

*“Strong educational training [with regard to] knowledge, skills and competencies related to [musculoskeletal] conditions and rehab need”*

In addition to strong professional and educational opportunities, descriptions of the use of evidence-based practice were commonly given as strengths within the education category of chiropractors delivering rehabilitation in health systems.

*“We base our rehab in evidence, but we understand the individuality of each patient”*

*“We follow guidelines within our profession – in Canada PEER, CCGI Centre for Effective Practice”*

Within the clinical practice category, interdisciplinary collaboration was commonly described as a strength for chiropractors delivering rehabilitation in health systems. Responses included mention of collaborations across multiple health professions.

*“Multiprofessional collaboration”*

*“Integrated communication between [chiropractors] and other health care providers ([general practitioners, physical therapists], occupational therapists)”*

Lastly, nearly all responses related to the category of leadership and policy highlighted positive advocacy and collaboration between chiropractic and rehabilitation professional organizations and governments. Global organizations (e.g., WHO, World Rehabilitation Alliance, WFC) and national bodies were described as providing strong leadership and maintaining positive relations with governmental agencies.

*“Advocacy through WFC”*

*“National associations meeting with health rebate companies”*

Representative quotes for other categories found within the strength domain of the SWOT framework are shown in Supplementary File 1.

### Weaknesses

Facilities/logistics (n = 20), finances (n = 15), education (n = 13), and leadership and policy (n = 13) factors were the most frequently described categories of weakness for the chiropractic profession. Within the facilities/logistics category, many respondents described a lack of integration of chiropractors into mainstream healthcare systems, including a weakness in the incorporation of electronic health records.

*“Lack of integration into funded primary care / mainstream health”*

*“No shared access to medical records”*

In addition, within the facilities/logistics category, respondents also frequently reported a modest chiropractic workforce as a weakness in rehabilitation in health systems. Respondents described not only a general workforce shortage but also multiple workforce shortages across geographically diverse regions, including LMICs.

*“Few chiropractors”*

*“Workforce shortages/inequity in low-mid income nations”*

Financial factors, such as socioeconomic challenges, socioeconomic inequalities, and challenges with payer reimbursement for services, were frequently described by respondents as a category of weakness.

*“Socioeconomic inequality making access to care difficult without monetary support”*

“*No reimbursement, expensive”*

Interestingly, chiropractic education was also described as a category of weakness. Multiple responses described a lack of chiropractors’ rehabilitation knowledge and a suboptimal number of chiropractic educational programs as weaknesses.

“*Lack of knowledge of chiropractic involvement in rehab”*

“*Lack of institutional programs”*

Similarly, leadership and policy factors were identified as a category of weakness, including the limited size of chiropractic organizations to advocate effectively politically and a lack of regulatory recognition across all regions of the world.

*“Not represented enough. Too small of a profession and not in all countries”*

*“Lack of legal recognition and regulation everywhere”*

Representative quotes for other categories found within the weakness domain of the SWOT framework are shown in Supplementary File 1.

### Opportunities

Facilities/logistics (n = 17), leadership and policy (n = 13), and education (n = 10) factors were the most frequent categories described as opportunities for delivery of rehabilitation in health systems. Respondents most prominently described opportunities within the facilities/logistics category, including opportunities for chiropractors to increase integration in health systems that need greater workforce capacity for musculoskeletal rehabilitation specialists.

*“Overwhelm of [general practitioner]/medical primary care – is an opportunity for us to take on [musculoskeletal] work from them”*

*“Increasing integration of [chiropractors] into hospital-based and primary care settings as equal members of health care teams”*

Respondents described opportunities within the leadership and policy category, with many responses suggesting national associations to increasingly advocate to governments for policy and health systems change.

*“Leverage regional and national [associations] to promote chiropractors and*

*rehab”*

*“Push associations lobby politicians what we have to offer”*

Lastly, respondents described the education category as an opportunity to increase research capacity and output related to chiropractors' rehabilitation delivery. Continuing professional development in rehabilitation and postgraduate education were seen as opportunities; specifically, residencies were reported as opportunities to enhance chiropractic rehabilitation delivery.

“*Advanced postgraduate education”*

“*Postgraduate residencies”*

Representative quotes for other categories found within the opportunities domain of the SWOT framework are shown in Supplementary File 1.

### Threats

Leadership and policy (n = 18), cultural (n = 15), and political (n = 13) factors were the most frequently described categories of threat. Within leadership and policy, many respondents described a lack of government mandates and/or policies that prioritize chiropractors as essential health workers for rehabilitation. To a lesser extent, the lack of professional organizational leadership to prioritize the delivery of rehabilitation by chiropractors was also identified as a threat.

*“Slow policy change to include and cover chiropractic services”*

*“Legislative changes not including all of the players at the trade and lack of patient / people input on [health care] needs.”*

Cultural factors were frequently described as a category of threat, with most responses concerning intra-professional threats focusing on non-evidence-based clinical behaviours of chiropractors or on non-evidence-based behaviours in their interactions with the public.

“*Non-evidence based practitioners.”*

“*Non-evidence based [chiropractors] on social media.”*

Lastly, political factors were frequently described as a category of threat to the delivery of rehabilitation by chiropractors. Nearly all the responses describing political threats concerned suboptimal inter-professional relationships or collaboration. “Tribalism” or “professional turf” wars were described as factors that influence inter-professional relationships and collaboration.

“*Professional tribalism to protect [money]”*

“*Interprofessional jealousy”*

Representative quotes for other categories found within the threats domain of the SWOT framework are shown in Supplementary File 1.

## Discussion

The overall findings from this study suggest that facilities/logistics, leadership and policy, and education were the most frequently reported categories across SWOT domains. These findings are generally comparable to findings of non-chiropractic centric studies investigating barriers and facilitators to rehabilitation services [20, 24]. Similarly, we found barriers and facilitators to the delivery of rehabilitation include a confluence of contextual factors (e.g., the physical, social, and attitudinal environment in which people live and conduct their lives) [24].

Interestingly, our findings did not include technology-related factors, such as telehealth. Htwe, et al. conducted a systematic review investigating barriers to accessibility to rehabilitation in LMICs [20]. The authors identified multiple barriers to telehealth, including financial, educational, community-based rehabilitation, social support, and clinical factors [20]. Though evidence for telehealth as a vehicle for the delivery of chiropractic services is limited, attention to telehealth has increased since the COVID-19 pandemic [25–27]. The literature suggests that barriers to chiropractors' engagement in telehealth as a form of service delivery include the reliability of technology, data privacy issues, therapeutic relationship building, digital health literacy, payment models, ambiguities in telehealth regulation, and chiropractors’ acceptance of telehealth [28–30]. Therefore, it is unsurprising that telehealth was not described in this study as a resource; regulations and clinician acceptability may remain low.

In this study, no responses were related to community-based rehabilitation programs. The WHO recently published community-based rehabilitation guidelines applicable to all disability groups [31]. Community-based rehabilitation is defined as “a strategy for community-based inclusive development which takes into account the principles of the Convention on the Rights of Persons with Disabilities, e.g. non-discrimination and the need to include all people with disabilities in development initiatives [31].” The lack of reporting of community-based rehabilitation as a barrier or facilitator to chiropractic delivery of rehabilitation in this study is concerning. It is possible that the lack of community-based rehabilitation in this study is due to a lack of awareness amongst the global chiropractic professional community. Further research is needed to better understand how community-based rehabilitation factors influence the delivery of rehabilitation by chiropractors worldwide.

Chiropractic integration in health systems was considered a strength within the facilities/logistics category, but also a weakness for chiropractors delivering rehabilitation in this study. This finding is not surprising, as evidence suggests that chiropractors are increasingly integrated in health systems throughout the world; however, widespread integration of chiropractors remains incomplete [2]. Most chiropractors remain in private practice in middle and upper-income communities at this time [32, 33].

Finances, such as costs to the patient and reimbursement costs to chiropractors, were reported as a category of weakness in chiropractic delivery of rehabilitation. Due to the lack of integration in public health systems, affordable access to chiropractic remains a problem, as literature suggests socioeconomic disparities in chiropractic utilization exist [12]. Lastly, cultural considerations, such as non-evidence-based behaviours, were commonly described as threats to the delivery of rehabilitation by chiropractors. This, too, is consistent with literature describing concerns with some individual chiropractors’ behaviours [34, 35]. A lack of clear and consistent professional identity within the chiropractic profession could contribute to the perpetuation of some chiropractors engaging in non-evidence-based behaviours [36].

Leadership and policy, and political factors such as legislative changes that often do not or are slow to recognize chiropractors as deliverers of rehabilitation, were seen as the greatest categories of threats. To aid in expanding the global rehabilitation workforce, chiropractic associations must continue to advocate at all levels of health systems, such as local and federal governments, to include chiropractic services in national health insurance and rehabilitation schemes. This is especially important in the context of chronic musculoskeletal conditions, such as back and neck pain, which have high disability and are associated with comorbidity [37–40]. Increased equitable access to chiropractic care, particularly for rural, underserved and ethnic minority populations, is essential [41–43]. The professional isolation of chiropractic in health systems can lead to fragmented care, duplication of services and poorer health outcomes for people who need rehabilitation. Chiropractors are increasingly included in rehabilitation plans and policy development globally, [44] though effective policy and advocacy at some regional and national levels are occurring more slowly. The findings from this study provide a blueprint for future, focused efforts for policymakers to overcome barriers and facilitators and work towards chiropractors participating as key contributors to meeting global rehabilitation needs.

### Future research

In this study, education was most frequently reported as a category of strength associated with the delivery of rehabilitation in health systems by chiropractors. Research is needed to investigate disability and rehabilitation curricula within chiropractic education programs worldwide. Understanding the frequency, quality, methods, and assessments of chiropractic education in disability and rehabilitation is needed to standardize teaching and accreditation expectations for disability and rehabilitation in chiropractic education programs. An enhancement of evidence-based education standards would additionally promote uniform expectations with the greater healthcare system and patients, and potentially reduce non-evidence-based behaviours.

Further, the exploration of unique and meaningful chiropractic clinical encounters is needed to verify existing information and validate the effectiveness of the delivery of rehabilitation by chiropractors for individuals living with musculoskeletal conditions with unmet health needs. Such research could be facilitated using existing data in registries and standards, increasing funding for observational research, and building chiropractic research on habilitation and rehabilitation. Lastly, context-specific implementation strategies need to be explored and tested to enhance chiropractic involvement in the delivery of rehabilitation in local, regional and global health systems. As the global rehabilitation workforce remains limited, [1] increased utilization of clinical specialists grounded in evidence-based rehabilitation, such as chiropractors, is needed.

### Strengths and limitations

This study had several strengths. The setting for this study was an intimate rehabilitation workshop at the 2025 WFC Biennial Congress. The WFC Biennial Congress commonly attracts a wide range of international chiropractic professional community members. Thus, this setting was an advantageous venue to potentially include a globally diverse sample. However, the demographic characteristics of our study sample, including its geographic representativeness, were not verified. Further, this study was strengthened by the completion of rigorous methodological processes. The study team conducted and clearly reported data preparation, data immersion, data analysis (e.g., coding matrix), and investigator reflexivity and positionality. Lastly, this study was strengthened using predefined SWOT categories and predefined barriers and facilitators that have been identified in rehabilitation delivery and utilization.

This study was limited by multiple factors. The nature of data collection in this study, through 4 structured question prompts, may limit the depth or richness of our data. Because data were collected during a time-limited workshop, participants were only given approximately 10-min for responses to each prompt. These likely favored brief responses, limited depth, and rich elaboration. Further research utilizing semi-structured interviews may allow for a greater depth and richness in data collected. In addition, this study included responses from 45 attendees at a rehabilitation workshop at a single chiropractic conference, where we did not collect demographic characteristics. Therefore, it is possible that respondents in this study may not be fully representative of all regions of the world.

## Conclusions

Respondents from a rehabilitation workshop at the 18th WFC Biennial Congress identified strengths, weaknesses, opportunities, and threats to the delivery of rehabilitation in chiropractors within health systems. The most frequent factors were facilities/logistics, leadership and policy, and education. While further study is needed to better understand unique barriers and facilitators in local, regional and global systems, immediate action is needed at all levels of chiropractic service delivery to promote chiropractors as key contributors to meeting global rehabilitation needs.

## Supplementary Information

Below is the link to the electronic supplementary material.


Supplementary Material 1.



Supplementary Material 2.



Supplementary Material 3.


## Data Availability

Data is available from the corresponding author upon reasonable request.
