# Supplementary material for: Barriers and facilitators to the delivery of rehabilitation in health systems by chiropractors: a SWOT analysis of perspectives from a world federation of chiropractic congress workshop
Source: Chiropr Man Therap. 2026 Jul 1;34:40. doi: 10.1186/s12998-026-00660-0 (PMC13525645; doi:10.1186/s12998-026-00660-0)
Supplement: Supplementary file 3 [file 12998_2026_660_MOESM3_ESM.docx]

**Supplementary File 1. Representative quotes for additional predefined categories of barriers and facilitators to the delivery of rehabilitation across each SWOT domain (Strengths, Weaknesses, Opportunities, Threats).**

| **Strengths** | |
| --- | --- |
| **Predefined category of barriers and facilitators to chiropractic delivery of rehabilitation in health systems** | **Representative quote** |
| Facilities/logistics | *“Easily accessible. Integrated in [health care] system”* |
| Registries and standards | *“Very strong evidence base that [chiropractors] can engage in rehab”* |
| Resources | *“An office that is already set up/has the equipment to offer one on one personalized rehab”* |
| Social support | *“Patient associations. Credibility.”* |
| Finances | *“Affordable”* |
| Political | *“Connections with the other health care providers”* |
| Cultural | *“Spine identity”* |
| **Weaknesses** | |
| **Predefined category of barriers and facilitators to chiropractic delivery of rehabilitation in health systems** | **Representative quote** |
| Political | *“Interprofessional conflicts”* |
| Social support | *“Patient prejudice to the chiropractors role”* |
| Cultural | *“’Unity’ identity”* |
| Clinical | *“ [clinical] skill deficit – need to pivot from existing approach with patients to areas do not know well”* |
| Resources | *“Lack of time”* |
| Registries and standards | *“Country [chiropractic] associations [do] not contribute to census and utilization data”* |
| **Opportunities** | |
| **Predefined category of barriers and facilitators to chiropractic delivery of rehabilitation in health systems** | **Representative quote** |
| Social support | *“Awareness of chiropractors as rehab experts”* |
| Political | *“Better interactions with [general practitioners]”* |
| Cultural | *“Early adopter on focus on rehab”* |
| Clinical | *“Collaborative management”* |
| Registries and standards | *“[Chiropractor] contributions to national workforce data”* |
| Finances | *“Get chiropractic recognized so insurance will pay”* |
| Resources | *“[able to provide] extra help for patients”* |
| **Threats** | |
| **Predefined category of barriers and facilitators to chiropractic delivery of rehabilitation in health systems** | **Representative quote** |
| Facilities/logistics | *“Few young [chiropractors]”* |
| Finances | *“Decreasing reimbursement for [chiropractic] services by 3^rd^ party payers”* |
| Social support | *“Lack of awareness of our existence”* |
| Education | *“Too few education centers for [training chiropractors]”* |
| Resources | *“Time”* |
| Clinical | *“Patient suffers a decline or negative outcome”* |
| Registries and standards | *“Standard of care”* |
